# Association of cumulative remnant cholesterol exposure with adverse outcomes after PCI in acute ST-segment elevation myocardial infarction

**DOI:** 10.3389/fcvm.2026.1842544

**Published:** 2026-08-26

**Authors:** Yazhao Sun, Feilong Shao, Xinxin Xu, Yayun Liu, Ya Ma

**Affiliations:** 1 Department of Cardiology, Cangzhou People's Hospital, Cangzhou, Hebei, China; 2 Department of General Practice, Huabei Petroleum Administration Bureau General Hospital, Cangzhou, Hebei, China; 3 Department of Ultrasound, Cangzhou People's Hospital, Cangzhou, Hebei, China

**Keywords:** cumulative exposure, major adverse cardiovascular and cerebrovascular events, percutaneous coronary intervention, remnant cholesterol, ST-segment elevation myocardial infarction

## Abstract

**Background:**

Remnant cholesterol (RC) has been associated with adverse cardiovascular outcomes; however, the relationship between cumulative RC exposure and clinical outcomes after percutaneous coronary intervention (PCI) in patients with ST-segment elevation myocardial infarction (STEMI) remains unclear. This study aimed to evaluate the association between cumulative remnant cholesterol (cumRC) and major adverse cardiovascular and cerebrovascular events (MACCEs) after PCI.

**Methods:**

This retrospective landmark study included 632 patients with STEMI who underwent successful PCI with drug-eluting stent implantation. RC was measured at baseline and at 2 and 4 years after PCI. cumRC was calculated as the area under the RC concentration-time curve over the fixed 4-year exposure period using the trapezoidal rule. The RC measurement obtained 4 years after PCI was defined as the landmark time point, followed by a 1-year outcome assessment. Multivariable logistic regression, subgroup analyses, and sensitivity analyses were performed.

**Results:**

During the 1-year post-landmark follow-up, 186 participants experienced at least one MACCE (29.4%). After multivariable adjustment, each 1 mmol/L·year increase in cumRC was associated with 36% higher odds of MACCEs (odds ratio [OR], 1.36; 95% confidence interval [CI], 1.15–1.61; *P* < 0.001). Compared with the lowest tertile, the second and third tertiles were associated with higher odds of MACCEs (second tertile: OR, 2.35; 95% CI, 1.47–3.76; *P* < 0.001; third tertile: OR, 2.71; 95% CI, 1.60–4.66; *P* < 0.001). No significant interactions were observed across the prespecified subgroups. The results remained consistent after separate adjustment for baseline low-density lipoprotein cholesterol (LDL-C) and high-density lipoprotein cholesterol (HDL-C), apolipoprotein B (ApoB), or baseline RC.

**Conclusion:**

Higher cumRC exposure was independently associated with higher odds of subsequent MACCEs in patients with STEMI after PCI. Prospective multicenter studies are needed to confirm this association and evaluate its clinical relevance.

## 1 Introduction

Cardiovascular diseases (CVD), particularly acute myocardial infarction (AMI), remain a leading cause of mortality worldwide, severely impacting individuals’ quality of life and life expectancy (1). Myocardial infarction (MI) is categorized into two types: non-ST elevation myocardial infarction (NSTEMI) and ST elevation myocardial infarction (STEMI). STEMI is typically triggered by complete occlusion of a coronary artery. Although advances in reperfusion therapy and pharmacological treatments have significantly reduced mortality in STEMI patients, the incidence of complications such as heart failure and arrhythmias has been steadily increasing, becoming important determinants of prognosis in these patients (2, 3). Therefore, early identification, prevention, and management of complications in MI patients remain critical issues in the treatment of cardiovascular diseases.

Atherosclerosis serves as the underlying pathological foundation of myocardial infarction (MI), with dyslipidemia playing a critical role in its progression. The hallmark of dyslipidemia is an elevation in low-density lipoprotein cholesterol (LDL-C) levels (4). Studies have demonstrated that reducing LDL-C levels significantly lowers the risk of recurrence and mortality in cardiovascular disease patients (5). A nationwide cohort study by Schubert et al., involving 40,607 patients, found that early reduction of LDL-C levels in MI patients could reduce all-cause mortality by 29% (6). For many years, the primary therapeutic targets for the prevention and treatment of dyslipidemia have focused on LDL-C levels (7). However, even when LDL-C levels are achieved to target, the risk of cardiovascular events remains, a phenomenon known as “residual risk” (8). Evidence suggests that changes in triglyceride-rich lipoproteins (TRLs) and their associated cholesterol, known as remnant cholesterol (RC), may play a key role in the progression of atherosclerosis (9, 10). TRL particles are highly atherogenic, capable of penetrating and accumulating within arterial walls. Due to their high cholesterol content, they are prone to forming foam cells and triggering inflammatory responses (11).

Qin et al. assessed RC levels in 2,312 patients with type 2 diabetes who underwent PCI. The results indicated that patients with higher baseline RC levels were more likely to experience restenosis within the stent (12). In a *post-hoc* analysis of the RACING trial, high RC levels were significantly associated with adverse clinical outcomes in atherosclerotic cardiovascular disease (ASCVD) patients receiving statin-based lipid-lowering therapy (13). Additionally, a study by Wang et al. found a significant correlation between RC levels and the occurrence of major adverse cardiovascular events (MACE) (14). Recent studies have shown that cumulative exposure to risk factors has a greater impact on adverse outcomes than a single measurement (15). However, most studies have focused on RC levels at a single time point, with few addressing the long-term cumulative exposure to RC and its role in cardiovascular risk. To date, the relationship between cumulative remnant cholesterol (cumRC) and clinical outcomes in STEMI patients undergoing PCI has not been thoroughly explored. Accordingly, this study aimed to evaluate the association between cumRC exposure and subsequent clinical outcomes in PCI-treated STEMI patients. The findings may provide evidence regarding the relationship between long-term RC burden and adverse outcomes in this population.

## 2 Methods

### 2.1 Study population

This retrospective landmark study included consecutive patients with STEMI who were admitted to Cangzhou People's Hospital between January 2018 and February 2019. STEMI was diagnosed according to the criteria established by the European Society of Cardiology (ESC), American College of Cardiology (ACC), American Heart Association (AHA), and the World Heart Federation (WHF) Myocardial Infarction Definition Working Group. All patients underwent successful PCI with drug-eluting stent (DES) implantation in the infarct-related artery. All procedures were performed by experienced interventional cardiologists using standard techniques, and guideline-recommended antiplatelet therapy was administered before and after PCI.

Patients were excluded if they had a history of myocardial infarction, previous PCI or coronary artery bypass grafting (CABG), cardiogenic shock, left ventricular ejection fraction (LVEF) < 30%, extreme obesity (body mass index [BMI] > 45 kg/m^2^), familial hypercholesterolemia, coagulation disorders, malignancies, chronic autoimmune diseases, congenital heart disease, severe metabolic disorders, acute or chronic infectious diseases, severe hepatic or renal dysfunction, experienced a MACCE or died before the 4-year landmark, incomplete RC measurements at baseline, 2 years, or 4 years after PCI, or incomplete post-landmark follow-up data.

**Figure 1:**
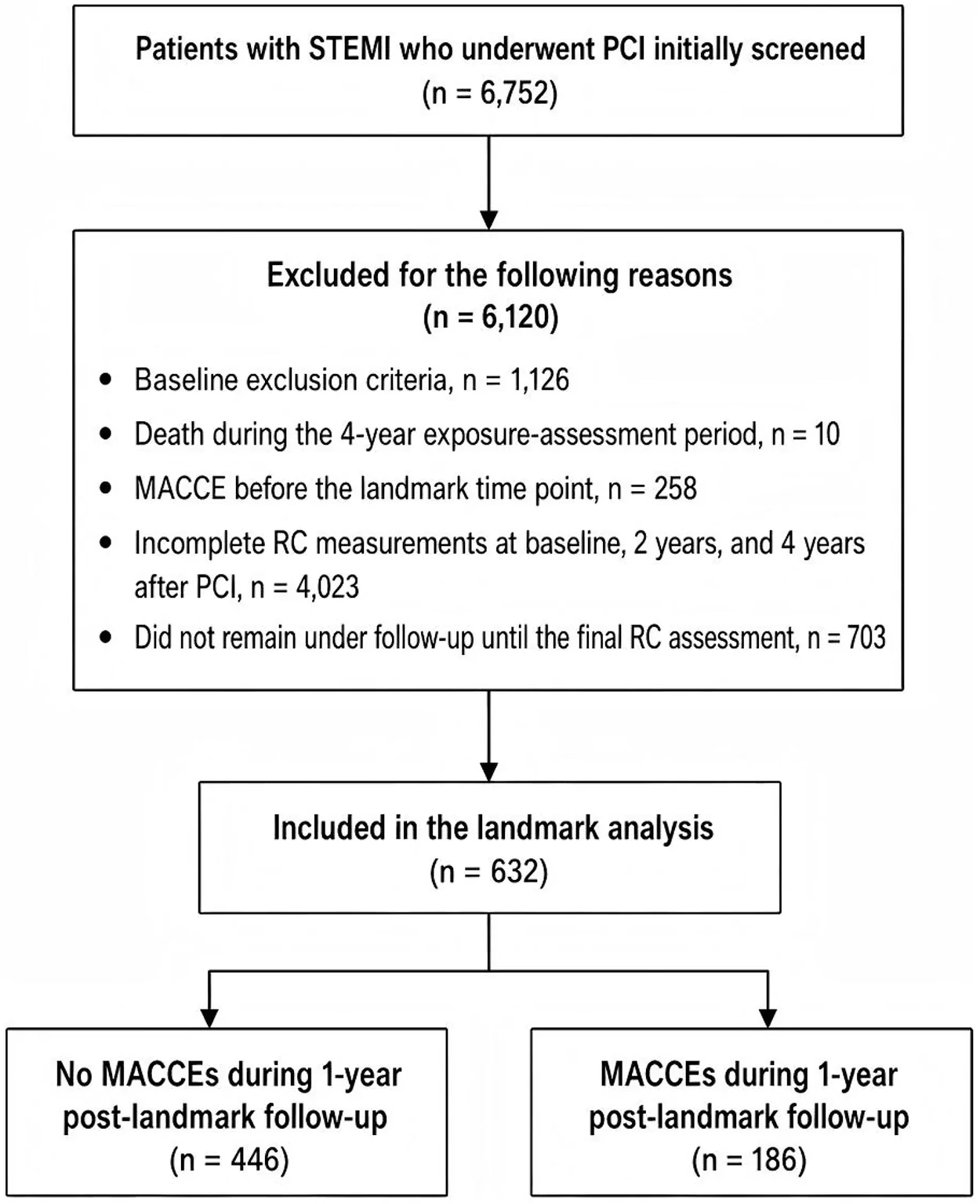
Flow diagram of participant selection. MACCEs, major adverse cardiovascular and cerebrovascular events; PCI, percutaneous coronary intervention; RC, remnant cholesterol; STEMI, ST-segment elevation myocardial infarction.

A total of 632 patients were included in the final analysis. This study was conducted in accordance with the Declaration of Helsinki and was approved by the Institutional Review Board of Cangzhou People's Hospital (K2023-154-03). Given the retrospective design and the use of anonymized clinical data, the requirement for informed consent was waived by the ethics committee. The detailed participant selection process is shown in Figure 1.

### 2.2 Data collection

Baseline clinical characteristics were retrospectively collected from the Hospital Information System (HIS) using standardized data collection forms. Demographic information, including age, sex, and BMI, and medical history, including coronary artery disease, stroke, heart failure, hypertension, diabetes, atrial fibrillation, anemia, chronic obstructive pulmonary disease (COPD), peripheral artery disease, and smoking status, were obtained. Laboratory data included white blood cell count, neutrophil count, lymphocyte count, monocyte count, fasting blood glucose, albumin, uric acid, creatinine, creatine kinase-MB (CK-MB), cardiac troponin I (TnI), B-type natriuretic peptide (BNP), total cholesterol (TC), triglycerides (TG), LDL-C, HDL-C, apolipoprotein A1 (ApoA1), apolipoprotein B (ApoB), baseline RC, and cumRC. Lipid parameters were measured in the hospital clinical laboratory using standardized procedures. TC and TG were measured using enzymatic methods, HDL-C and LDL-C using direct homogeneous assays, and ApoA1 and ApoB using immunoturbidimetric assays. Baseline RC was calculated as TC minus HDL-C minus LDL-C. Echocardiographic parameters, including LVEF, were also collected. Procedural characteristics, including vascular access approach, number of diseased vessels, number of treated vessels, and number of implanted stents, were obtained from PCI records. Medication information at discharge, including aspirin, ticagrelor/clopidogrel, β-blockers, angiotensin-converting enzyme inhibitors (ACEI), and statins, was recorded. All data were extracted by trained investigators and reviewed for completeness and accuracy before statistical analysis.

### 2.3 Definition of cumRC

The primary exposure of interest was cumRC. RC was calculated as TC minus HDL-C and LDL-C. RC was measured at three prespecified time points relative to the index PCI: at baseline (RC0), 2 years after PCI (RC1), and 4 years after PCI (RC2). For patients with complete RC measurements at all three time points, cumRC was calculated as the area under the RC concentration-time curve over the fixed 4-year exposure assessment period using the trapezoidal rule as follows: cumRC = [(RC₀ + RC₁)/2] × 2 years + [(RC₁ + RC₂)/2] × 2 years.

The resulting cumRC represented cumulative RC exposure during the first 4 years after PCI and was expressed in mmol/L·year. Because the measurement schedule was prespecified, the interval between consecutive measurements was fixed at 2 years for all participants. Patients without complete RC measurements at all three time points were excluded, and no interpolation or imputation of missing RC values was performed.

### 2.4 Follow-up and outcomes

The RC2 measurement obtained 4 years after the index PCI was defined as the landmark time point. Only patients who were alive and free of MACCEs at the landmark time point were included in the post-landmark outcome analysis. Participants were followed for 1 year after the landmark time point through hospital readmission records, outpatient visits, online medical services, WeChat, or telephone contact. The primary outcome was the occurrence of MACCEs, defined as a composite of all-cause mortality, MI, target vessel revascularization, unstable angina requiring hospitalization, heart failure, and ischemic stroke. For participants who experienced more than one component event, only the first event was included in the primary analysis. MI was defined according to the fourth universal definition of myocardial infarction, based on clinical presentation, electrocardiographic changes, and elevated cardiac biomarkers. Target vessel revascularization referred primarily to repeat PCI involving the culprit vessel or one of its major branches. Unstable angina was defined as new or worsening angina requiring hospitalization. Heart failure was diagnosed based on clinical symptoms and signs, supported by biomarkers such as BNP. Ischemic stroke was defined by acute neurological deficits confirmed by CT or MRI.

### 2.5 Statistical analyses

Continuous variables were presented as mean ± SD or median and IQR, as appropriate, and categorical variables were presented as numbers and percentages. Differences between groups were assessed using the independent-samples t-test, Mann–Whitney U test, chi-square test, or Fisher's exact test, as appropriate. The association between cumRC and MACCEs was evaluated using logistic regression models, with ORs and 95% CIs reported. cumRC was analyzed as both a continuous variable and a categorical variable according to tertiles. Model 1 was unadjusted. Model 2 was adjusted for age, sex, BMI, hypertension, diabetes, smoking history, atrial fibrillation, prior stroke, LVEF, serum creatinine, multivessel coronary disease, and treatment of two or more vessels. These covariates were selected *a priori* based on their clinical relevance and potential confounding effects. VIFs were calculated for all adjusted models to assess multicollinearity. A VIF >5 was considered indicative of potentially problematic multicollinearity. Subgroup analyses were adjusted for the covariates included in Model 2, except for the variable defining the corresponding subgroup. Interactions between cumRC and subgroup variables were evaluated by including multiplicative interaction terms in the regression models. Sensitivity analyses using alternative lipid-adjustment strategies were also performed to assess the robustness of the findings. To assess the incremental model performance of cumRC while minimizing multicollinearity, baseline RC, baseline LDL-C and HDL-C, and baseline ApoB were evaluated in separate nested models. Model performance before and after the addition of cumRC was compared using the likelihood ratio test, Akaike information criterion (AIC), and C-statistic. All statistical analyses were performed using R software version 4.4.2. All tests were two-sided, and *P* < 0.05 was considered statistically significant.

## 3 Results

### 3.1 Participant characteristics

A total of 632 participants were included in the study, of whom 446 did not experience MACCEs and 186 experienced at least one MACCE during the 1-year post-landmark follow-up. Baseline characteristics according to MACCE status are presented in Table 1. Compared with participants without MACCEs, those with MACCEs had a higher prevalence of diabetes and atrial fibrillation, a higher BMI, and higher levels of uric acid, serum creatinine, CK-MB, TnI, BNP, TC, TG, LDL-C, ApoB, baseline RC, and cumRC. ACEI use at discharge was less frequent among participants with MACCEs. No significant between-group differences were observed in the other baseline characteristics.

**Table 1:** Baseline characteristics according to MACCE status.

Characteristics	Overall (*n* = 632)	Without MACCEs (*n* = 446)	With MACCEs (*n* = 186)	*P* value
Demographic characteristics				
Age, years	66.00 (56.00–72.50)	65.00 (56.00–72.00)	66.00 (57.00–74.00)	0.092
Male sex, *n* (%)	425 (67.25%)	299 (67.04%)	126 (67.74%)	0.864
BMI, kg/m^2^	25.45 ± 3.40	25.24 ± 3.47	25.95 ± 3.17	0.013
Medical history and comorbidities				
History of coronary artery disease, *n* (%)	120 (18.99%)	89 (19.96%)	31 (16.67%)	0.337
History of stroke, *n* (%)	50 (7.91%)	39 (8.74%)	11 (5.91%)	0.230
History of heart failure, *n* (%)	88 (13.92%)	58 (13.00%)	30 (16.13%)	0.301
Hypertension, *n* (%)	339 (53.64%)	237 (53.14%)	102 (54.84%)	0.696
Diabetes, *n* (%)	128 (20.25%)	81 (18.16%)	47 (25.27%)	0.043
Atrial fibrillation, *n* (%)	20 (3.16%)	9 (2.02%)	11 (5.91%)	0.011
Anemia, *n* (%)	67 (10.60%)	48 (10.76%)	19 (10.22%)	0.839
COPD, *n* (%)	6 (0.95%)	3 (0.67%)	3 (1.61%)	0.267
Peripheral artery disease, *n* (%)	24 (3.80%)	16 (3.59%)	8 (4.30%)	0.669
Smoking history, *n* (%)	442 (69.94%)	307 (68.83%)	135 (72.58%)	0.349
Cardiac function				
LVEF, %	53.00 (50.00–59.00)	53.00 (50.00–59.00)	53.00 (49.00–59.00)	0.086
Laboratory parameters				
White blood cell count, ×10⁹/L	8.58 (6.90–10.83)	8.58 (6.94–10.89)	8.61 (6.50–10.60)	0.880
Neutrophil count, ×10⁹/L	6.58 (4.74–8.73)	6.56 (4.87–8.83)	6.69 (4.67–8.48)	0.848
Lymphocyte count, ×10⁹/L	1.37 (0.97–1.89)	1.35 (1.00–1.90)	1.41 (0.94–1.86)	0.857
Monocyte count, ×10⁹/L	0.42 (0.32–0.55)	0.42 (0.32–0.55)	0.42 (0.33–0.57)	0.367
Fasting blood glucose, mmol/L	6.57 (5.57–8.51)	6.52 (5.57–8.33)	6.61 (5.48–8.72)	0.847
Albumin, g/L	40.53 ± 3.93	40.43 ± 3.96	40.78 ± 3.87	0.317
Uric acid, μmol/L	309.00 (253.50–368.05)	299.50 (245.00–357.00)	328.65 (270.00–415.80)	<0.001
Serum creatinine, μmol/L	67.00 (55.95–80.00)	66.00 (55.00–77.10)	70.00 (58.00–87.00)	0.020
CK-MB, ng/mL	15.94 (12.09–20.46)	15.58 (12.00–19.96)	16.48 (12.60–22.61)	0.023
TnI, pg/mL	523.50 (131.50–2743.00)	472.00 (120.00–2470.00)	713.50 (183.00–3543.00)	0.034
BNP, pg/mL	189.15 (71.15–634.75)	176.35 (69.40–590.00)	212.60 (77.40–909.70)	0.036
TC, mmol/L	4.62 ± 1.09	4.54 ± 1.06	4.80 ± 1.13	0.008
TG, mmol/L	1.34 (0.92–1.83)	1.28 (0.87–1.78)	1.49 (1.03–1.94)	0.012
HDL-C, mmol/L	1.09 (0.92–1.31)	1.10 (0.92–1.32)	1.06 (0.91–1.28)	0.238
LDL-C, mmol/L	2.82 ± 0.96	2.76 ± 0.93	2.95 ± 1.03	0.034
ApoA1, g/L	1.21 (1.04–1.43)	1.21 (1.03–1.42)	1.21 (1.04–1.44)	0.776
ApoB, g/L	0.88 ± 0.27	0.86 ± 0.27	0.90 ± 0.27	0.006
Baseline RC, mmol/L	0.56 (0.36–0.84)	0.54 (0.35–0.82)	0.59 (0.38–0.94)	0.009
cumRC, mmol/L·year	2.48 (1.93–3.24)	2.35 (1.81–3.07)	2.81 (2.15–3.77)	<0.001
Procedural characteristics				
Radial access, *n* (%)	588 (93.04%)	412 (92.38%)	176 (94.62%)	0.312
Number of diseased vessels, *n* (%)				0.206
One vessel	246 (38.92%)	165 (37.00%)	81 (43.55%)	
Two vessels	260 (41.14%)	193 (43.27%)	67 (36.02%)	
Three vessels	126 (19.94%)	88 (19.73%)	38 (20.43%)	
Number of implanted stents ≥2, *n* (%)	399 (63.13%)	287 (64.35%)	112 (60.22%)	0.326
Number of treated vessels ≥2, *n* (%)	181 (28.64%)	120 (26.91%)	61 (32.80%)	0.136
Medications at discharge				
Aspirin, *n* (%)	626 (99.05%)	442 (99.10%)	184 (98.92%)	0.833
Ticagrelor or clopidogrel, *n* (%)	614 (97.15%)	431 (96.64%)	183 (98.39%)	0.228
β-blockers, *n* (%)	383 (60.60%)	264 (59.19%)	119 (63.98%)	0.262
ACEI, *n* (%)	375 (59.34%)	277 (62.11%)	98 (52.69%)	0.028
Statins, *n* (%)	615 (97.31%)	431 (96.64%)	184 (98.92%)	0.105

ACEI, angiotensin-converting enzyme inhibitor; ApoA1, apolipoprotein A1; ApoB, apolipoprotein B; BMI, body mass index; BNP, B-type natriuretic peptide; CI, confidence interval; CK-MB, creatine kinase-MB; COPD, chronic obstructive pulmonary disease; cumRC, cumulative remnant cholesterol; HDL-C, high-density lipoprotein cholesterol; IQR, interquartile range; LDL-C, low-density lipoprotein cholesterol; LVEF, left ventricular ejection fraction; MACCEs, major adverse cardiovascular and cerebrovascular events; RC, remnant cholesterol; SD, standard deviation; TC, total cholesterol; TG, triglycerides; TnI, cardiac troponin I.

### 3.2 Association between cumRC and MACCEs

During the 1-year post-landmark follow-up, 186 of the 632 participants experienced at least one MACCE. When classified according to the first qualifying event, these included 1 all-cause death, 11 MIs, 56 target vessel revascularizations, 93 hospitalizations for unstable angina, 15 heart failure events, and 10 ischemic strokes. Each 1 mmol/L·year increase in cumRC was associated with 41% higher odds of MACCEs in the unadjusted model (OR, 1.41; 95% CI, 1.23–1.62; *P* < 0.001) and 36% higher odds after multivariable adjustment (OR, 1.36; 95% CI, 1.15–1.61; *P* < 0.001) (Table 2). Similar results were observed in the tertile analysis. Compared with T1, participants in T2 and T3 had higher odds of MACCEs in the unadjusted model (T2: OR, 2.61; 95% CI, 1.59–4.34; *P* < 0.001; T3: OR, 2.78; 95% CI, 1.78–4.40; *P* < 0.001) and in Model 2 (T2: OR, 2.35; 95% CI, 1.47–3.76; *P* < 0.001; T3: OR, 2.71; 95% CI, 1.60–4.66; *P* < 0.001). The VIFs for cumRC, age, sex, BMI, hypertension, diabetes, smoking history, atrial fibrillation, prior stroke, LVEF, serum creatinine, multivessel coronary disease, and treatment of two or more vessels were 1.28, 1.31, 1.16, 1.22, 1.41, 1.35, 1.27, 1.19, 1.14, 1.24, 1.29, 1.46, and 1.52, respectively. The maximum VIF was 1.52, and all values were below the prespecified threshold of 5, indicating no problematic multicollinearity in Model 2.

**Table 2:** Association between cumRC and MACCEs.

Exposure	Model 1 OR (95% CI)	*P* value	Model 2 OR (95% CI)	*P* value
cumRC, per 1 mmol/L·year increase	1.41 (1.23–1.62)	<0.001	1.36 (1.15–1.61)	<0.001
Tertiles of cumRC				
T1	Reference	–	Reference	–
T2	2.61 (1.59–4.34)	<0.001	2.35 (1.47–3.76)	<0.001
T3	2.78 (1.78–4.40)	<0.001	2.71 (1.60–4.66)	<0.001

cumRC was analyzed per 1 mmol/L·year increase as a continuous variable and was also categorized into tertiles (T1–T3). Model 1 was unadjusted. Model 2 was adjusted for age, sex, BMI, hypertension, diabetes, smoking history, atrial fibrillation, prior stroke, LVEF, serum creatinine, multivessel coronary disease, and treatment of two or more vessels.

CI, confidence interval; OR, odds ratio; cumRC, cumulative remnant cholesterol; MACCEs, major adverse cardiovascular and cerebrovascular events; BMI, body mass index; LVEF, left ventricular ejection fraction.

### 3.3 Subgroup analysis

Subgroup analyses were performed to assess whether the association between cumRC and MACCEs varied across clinical characteristics. Higher cumRC was associated with higher odds of MACCEs in most subgroups (Table 3). The associations were not statistically significant among participants aged <60 years, those without hypertension, or those with LDL-C < 1.80 mmol/L, although all ORs were greater than 1. No significant interaction was observed for age, sex, BMI, hypertension, diabetes, or LDL-C (all *P* for interaction >0.05). Given the multiple comparisons and limited sample sizes in some strata, these findings should be interpreted as exploratory.

**Table 3:** Subgroup analysis of the association between cumRC and MACCEs.

Characteristic	Group	OR (95% CI)	*P* value	*P* for interaction
Age	<60 years (*n* = 195)	1.29 (0.96–1.74)	0.093	0.673
	≥60 years (*n* = 437)	1.39 (1.16–1.66)	<0.001	
Sex	Male (*n* = 425)	1.31 (1.07–1.60)	0.009	0.514
	Female (*n* = 207)	1.47 (1.11–1.95)	0.007	
BMI	<24 kg/m^2^ (*n* = 227)	1.43 (1.06–1.93)	0.019	0.663
	≥24 kg/m^2^ (*n* = 405)	1.32 (1.08–1.61)	0.006	
Hypertension	No (*n* = 293)	1.18 (0.94–1.49)	0.159	0.095
	Yes (*n* = 339)	1.57 (1.23–2.00)	<0.001	
Diabetes	No (*n* = 504)	1.25 (1.05–1.48)	0.011	0.114
	Yes (*n* = 128)	1.87 (1.17–2.99)	0.009	
LDL-C	<1.80 mmol/L (*n* = 79)	1.20 (0.72–2.01)	0.486	0.595
	≥1.80 mmol/L (*n* = 553)	1.39 (1.17–1.65)	<0.001	

Each stratified model was adjusted for the covariates included in Model 2, except for the variable used to define the corresponding subgroup.

BMI, body mass index; CI, confidence interval; cumRC, cumulative remnant cholesterol; LDL-C, low-density lipoprotein cholesterol; MACCEs, major adverse cardiovascular and cerebrovascular events; OR, odds ratio.

### 3.4 Sensitivity analyses

Sensitivity analyses yielded results consistent with the primary analysis. Each 1 mmol/L·year increase in cumRC remained associated with higher odds of MACCEs after additional adjustment for baseline LDL-C and HDL-C (OR, 1.34; 95% CI, 1.13–1.59; *P* < 0.001), baseline ApoB (OR, 1.32; 95% CI, 1.11–1.57; *P* = 0.002), and baseline RC (OR, 1.27; 95% CI, 1.05–1.54; *P* = 0.014), although the estimate was attenuated after adjustment for baseline RC. These findings were consistent with the primary analysis.

### 3.5 Incremental model performance of cumRC

The incremental model performance of cumRC is presented in Table 4. Adding cumRC to the model containing baseline RC reduced the AIC from 743.2 to 739.1 and increased the C-statistic from 0.676 to 0.690 (likelihood ratio *P* = 0.014). When cumRC was added to the model containing baseline LDL-C and HDL-C, the AIC decreased from 746.0 to 736.9, while the C-statistic increased from 0.671 to 0.691 (likelihood ratio *P* < 0.001). Similarly, adding cumRC to the model containing baseline ApoB reduced the AIC from 744.5 to 737.0 and increased the C-statistic from 0.674 to 0.689 (likelihood ratio *P* = 0.002). Overall, the addition of cumRC improved model fit, although the increases in discrimination were modest.

**Table 4:** Incremental model performance of cumRC.

Baseline lipid parameter(s)	AIC, before/after adding cumRC	C-statistic, before/after adding cumRC	Likelihood ratio *P* value
Baseline RC	743.2/739.1	0.676/0.690	0.014
Baseline LDL-C and HDL-C	746.0/736.9	0.671/0.691	<0.001
Baseline ApoB	744.5/737.0	0.674/0.689	0.002

The basic clinical model included age, sex, BMI, hypertension, diabetes, smoking history, atrial fibrillation, prior stroke, LVEF, serum creatinine, multivessel coronary disease, and treatment of two or more vessels.

AIC, Akaike information criterion; ApoB, apolipoprotein B; BMI, body mass index; cumRC, cumulative remnant cholesterol; HDL-C, high-density lipoprotein cholesterol; LDL-C, low-density lipoprotein cholesterol; LVEF, left ventricular ejection fraction; RC, remnant cholesterol.

## 4 Discussion

This landmark study demonstrated that higher cumRC exposure during the first 4 years after PCI was independently associated with higher odds of MACCEs during the subsequent 1-year follow-up in patients with STEMI undergoing first PCI. The association remained significant after adjustment for prespecified clinical covariates. Consistent findings were observed when cumRC was analyzed as a continuous variable or according to tertiles. No significant interactions were identified in the prespecified subgroup analyses. The results also remained robust in sensitivity analyses using alternative lipid-adjustment strategies. Nested model comparisons further showed that adding cumRC improved overall model fit beyond models containing baseline RC, baseline LDL-C and HDL-C, or baseline ApoB, although the improvements in discrimination were modest.

LDL-C remains the primary therapeutic target for ASCVD prevention and management. LDL particles and other ApoB-containing lipoproteins participate in cholesterol transport and play a central role in atherosclerosis development (16). These particles can cross the endothelial barrier, become retained in the arterial extracellular matrix, and undergo oxidative modification, leading to endothelial injury, inflammatory activation, and plaque formation (17). Clinical trials have demonstrated that LDL-C lowering significantly reduces cardiovascular events (18, 19). However, the relationship between LDL-C and cardiovascular risk is not determined solely by current LDL-C levels. Ference et al. demonstrated that cumulative LDL-C exposure over time is strongly associated with ASCVD risk, emphasizing the importance of lifelong lipid burden (15). Previous studies have also shown that prolonged exposure to elevated LDL-C is closely related to ASCVD development (20, 21). In patients with established cardiovascular disease receiving lipid-lowering therapy, higher LDL-C levels remain associated with increased recurrent cardiovascular events (22). Moreover, Navarese et al. reported that cardiovascular mortality progressively decreased with further LDL-C reduction among patients with elevated baseline LDL-C levels in a meta-analysis involving 34 randomized clinical trials and 270,288 participants (23).

Despite the benefits of intensive LDL-C reduction, residual cardiovascular risk remains in patients with ASCVD. Previous pharmacological interventions aimed at increasing HDL-C levels have failed to consistently reduce cardiovascular events (24). Therefore, attention has shifted toward triglyceride-rich lipoproteins (TRLs) and their remnants. RC represents the cholesterol content carried by TRLs and reflects cholesterol contained in remnant particles beyond LDL-C and HDL-C (25). Recent studies have demonstrated that RC contributes to residual ASCVD risk beyond LDL-C and other traditional lipid parameters (26, 27). Elevated RC levels have been associated with increased MACE risk among patients with type 2 diabetes even after adjustment for potential confounders (28). Furthermore, RC has been associated with cardiovascular risk in individuals without established CVD independent of traditional risk factors, LDL-C, and ApoB levels (29). Delialis et al. further emphasized the association of RC with cardiovascular risk and clinical events (30).

The association between RC and cardiovascular outcomes may involve multiple biological pathways. RC particles can accumulate within the arterial wall and are readily taken up by macrophages, promoting foam cell formation and accelerating atherosclerotic progression (31–34). In addition, RC accumulation is associated with vascular inflammation and endothelial dysfunction, which may contribute to plaque instability and lesion progression (35). RC also exhibits pro-inflammatory properties similar to oxidized LDL, and triglyceride-rich remnant particles may induce oxidative stress and endothelial injury through lipid metabolism-related pathways (36, 37). Compared with LDL-C, RC has been reported to show stronger associations with arterial stiffness and cardiovascular risk assessment (38). Genetic studies have further demonstrated that elevated RC levels are associated with coronary artery disease and myocardial infarction independent of LDL-C levels (39, 40).

Compared with a single RC measurement, cumulative exposure may better reflect the long-term burden of RC. Previous studies have examined the association between cumRC exposure and cardiovascular outcomes. Dai et al. reported that higher cumRC exposure was independently associated with cardiovascular events in young and middle-aged adults during a 30-year follow-up period (41). Zhao et al. followed 5,372 participants for 3 years and found a positive association between cumRC and CVD risk, with each unit increase in cumRC associated with a 13% higher risk of CVD (42). Xiao et al. further reported that elevated cumRC exposure was independently associated with cardiovascular events in elderly patients with ASCVD during a 9-year follow-up period (43). Our study extends these findings by demonstrating that higher cumRC exposure was associated with higher odds of MACCEs in patients with STEMI undergoing first PCI. Unlike previous studies conducted mainly in general populations or patients with chronic ASCVD, our study focused on a high-risk population after acute coronary intervention. In separate sensitivity analyses, the association remained significant after additional adjustment for baseline LDL-C and HDL-C, ApoB, or baseline RC, suggesting that cumRC may capture information not fully reflected by a single baseline lipid measurement. However, these findings establish an association and do not demonstrate incremental predictive value or clinical utility.

## 5 Limitations

Several limitations should be acknowledged. First, cumRC was estimated from three serial calculated RC measurements using the trapezoidal rule rather than from directly measured remnant lipoprotein cholesterol. Measurement variability at individual time points may therefore have affected the cumulative exposure estimate. Second, the composite endpoint included events of different clinical severity and combined relatively objective events with outcomes that may be more dependent on clinical judgment. Although the component-specific event counts were reported, only 22 hard endpoint events, comprising all-cause mortality, MI, and ischemic stroke, occurred. This limited the feasibility of a stable multivariable analysis using the restricted hard composite endpoint. Third, although the primary model included a nonredundant set of clinical covariates and showed no problematic multicollinearity, residual confounding cannot be excluded because of the observational design. In addition, baseline RC is mathematically related to cumRC; therefore, the attenuated estimate in the sensitivity analysis adjusted for baseline RC should be interpreted cautiously. Fourth, detailed longitudinal information on statin type and intensity, changes in lipid-lowering therapy, use of additional lipid-lowering agents, and medication adherence was not systematically available. Statin use at discharge alone could not adequately represent treatment exposure during the 4-year cumRC assessment period. Fifth, the retrospective landmark design may have introduced selection bias and survivor bias because only patients who survived, remained free of MACCEs during the first 4 years after PCI, and completed all required RC measurements were included. Consequently, the final cohort may represent a relatively healthier and more closely followed subgroup, whereas patients at higher risk of early death or recurrent cardiovascular events were not represented in the post-landmark analysis. The findings may therefore not be generalizable to all patients with STEMI after PCI, particularly those who experienced MACCEs or died before the 4-year landmark. This selection process may also have affected the magnitude of the observed association, although the direction of the potential bias cannot be determined from the present data. The 4-year landmark time point was determined by the available serial RC measurement schedule and the structure of the retrospective dataset, rather than by an established biological threshold or a clinically validated optimal exposure duration. Because alternative landmark time points and cumulative exposure windows were not evaluated, it remains uncertain whether the observed association would be consistent over shorter or longer exposure periods. The single-center setting may also limit the generalizability of the findings. Moreover, the 1-year post-landmark follow-up period may have been insufficient to capture longer-term outcomes, and the underlying biological mechanisms were not investigated. Multicenter prospective studies with more frequent serial RC measurements, comparisons across different exposure windows, and longer follow-up are needed to validate these findings.

## 6 Conclusion

In conclusion, higher cumRC exposure was independently associated with higher odds of subsequent MACCEs in patients with STEMI after first PCI. Prospective multicenter studies are needed to confirm this association and evaluate its clinical relevance before cumRC can be considered for clinical risk assessment.

## Data Availability

The original contributions presented in the study are included in the article/Supplementary Material, further inquiries can be directed to the corresponding authors.
